# Mining small RNA structure elements in untranslated regions of human and mouse mRNAs using structure-based alignment

**DOI:** 10.1186/1471-2164-9-189

**Published:** 2008-04-25

**Authors:** Mugdha Khaladkar, Jianghui Liu, Dongrong Wen, Jason TL Wang, Bin Tian

**Affiliations:** 1 Department of Biochemistry and Molecular Biology, New Jersey Medical School, University of Medicine and Dentistry of New Jersey, Newark, NJ 07103, USA; 2 Department of Computer Science, New Jersey Institute of Technology, Newark, NJ 07102, USA

## Abstract

**Background:**

UnTranslated Regions (UTRs) of mRNAs contain regulatory elements for various aspects of mRNA metabolism, such as mRNA localization, translation, and mRNA stability. Several RNA stem-loop structures in UTRs have been experimentally identified, including the histone 3' UTR stem-loop structure (HSL3) and iron response element (IRE). These stem-loop structures are conserved among mammalian orthologs, and exist in a group of genes encoding proteins involved in the same biological pathways. It is not known to what extent RNA structures like these exist in all mammalian UTRs.

**Results:**

In this paper we took a systematic approach, named GLEAN-UTR, to identify small stem-loop RNA structure elements in UTRs that are conserved between human and mouse orthologs and exist in multiple genes with common Gene Ontology terms. This approach resulted in 90 distinct RNA structure groups containing 748 structures, with HSL3 and IRE among the top hits based on conservation of structure.

**Conclusion:**

Our result indicates that there may exist many conserved stem-loop structures in mammalian UTRs that are involved in coordinate post-transcriptional regulation of biological pathways.

## Background

RNA *cis *elements residing in the UnTranslated Regions (UTRs) of mRNAs have been shown to play various roles in post-transcriptional gene regulation, including mRNA localization, translation, and mRNA stability [1-4]. The function of a *cis *element can be attributable to its primary sequence or structure. For simplicity, they are called sequence elements and structure elements, respectively. Well-known sequence elements include AU-rich elements (ARE), which contain one or several tandem AUUUA sequences and are involved in modulation of mRNA stability [5,6], and miRNA target sites, which base-pair with their cognate miRNA molecules and are involved in the regulation of translation or mRNA stability [7,8]. Well-characterized structure elements include Internal Ribosome Entry Site (IRES) [9] and Iron Response Element (IRE) [10] in the 5' UTR, Selenocysteine Insertion Sequence (SECIS) [11], IRE, and histone 3' UTR stem-loop structure (HSL3) [12] in the 3' UTR. Each element type exists in multiple genes, and thus can be considered as an RNA motif (similar to the concept of protein motif). IRE and HSL3 elements are highly similar to one another within each type; some divergence has been reported for SECIS [11]; and there is no extensive similarity in primary sequence or secondary structure among IRES elements [9]. These characteristics reflect the ways that the RNA structures function. In addition, various gene-specific structure elements in 5' or 3'UTRs have been shown to play roles in various aspects of RNA metabolism [1].

Functional RNA sequence elements in the human genome have been heavily studied in recent years, including elements responsible for pre-mRNA splicing, polyadenylation, and miRNA target sites [13-17]. In contrast, RNA structure elements have been investigated to a much lesser extent, partly due to the difficulties in accurately predicting and aligning RNA structures, and assessing false discovery rate (FDR). Recent developments of genome-wide prediction of RNA structures based on aligned genomes [18,19] or unalignable regions [20] have resulted in large numbers of conserved RNA structures. On one hand, all methods reported high potential FDR. On the other hand, these results vary from one another in coverage, indicating that there may exist even more structures to be discovered. Here, we took an approach that is not based on genome alignments, dubbed GLEAN-UTR (grouping by structural distanceand ontology for RNA elements in UTRs) to uncover conserved RNA structures in UTRs. We focused on small stem-loop structures. We compared folded RNA structures in UTR sequences for orthologous genes by our RNA structure alignment tool RSmatch [21]. Similar orthologous structures were then compared in an all-against-all fashion to derive RNA structure groups. Using cluster analysis and Gene Ontology (GO) information, we identified RNA structures that exist in multiple genes that share common biological pathways. For 10,448 human genes which were analyzed, we obtained 90 RNA structure groups, containing 748 distinct RNA structures in 3' or 5' UTRs from 698 genes. HSL3 and IRE are among the top hits based on conservation of structure. Using a randomized data set, we estimated FDR of 15% for all the structures. About 12% of the structures overlap genomic regions identified by other whole-genome wide studies for RNA structures. This bioinformatics study lays groundwork for future wet lab examination of putative conserved RNA structure elements in human and mouse UTRs.

## Results

### Mining RNA structure elements in UTRs

**Figure 1:**
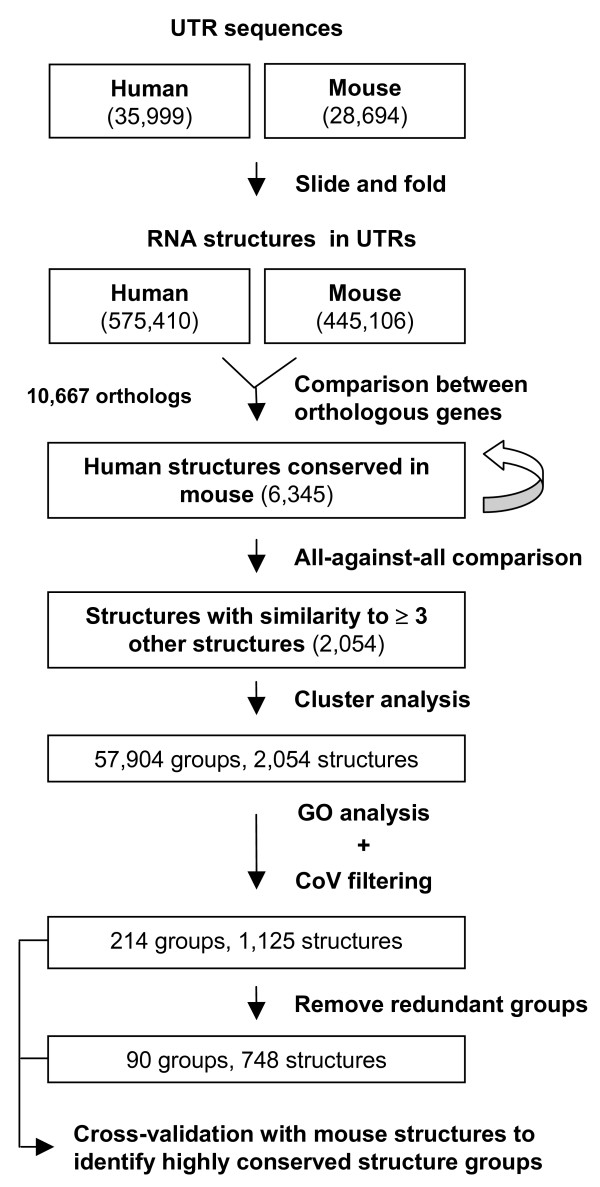
**GLEAN-UTR**. This flowchart presents the overall methodology used in this study. The numbers of RNA structures and structure groups are indicated at each step.

We wanted to identify functional structure elements in human UTRs. Previous studies have used aligned vertebrate genomes to predict conserved structures in the whole genome [18,19]. However, a recent report indicated that many human genome regions containing RNA structures cannot be aligned with the mouse genome [20]. This suggests that reliance on genome alignments containing divergent species, such as human and fish, may result in many false negatives. This situation can be exacerbated for UTRs, which typically do not exhibit large rates of sequence conservation. To explore approaches other than using aligned genomes, we designed a method, named GLEAN-UTR, which is based on the rationale that there exist structure elements in 5' and 3' UTRs that are encoded by a group of genes involved in the same biological pathways, similar to IRE and HSL3 structures (see Additional file 1). We applied the method to human and mouse UTRs. Figure 1 shows the overall design and procedure of this method.

We first extracted UTR sequences from NCBI RefSeq sequences. We then used a "slide and fold" method to construct RNA structures in 5' and 3' UTRs (see Methods for detail). With this method, subsequences in UTRs, 100 nucleotides (nt) long or less, were folded according to thermodynamic properties using the Vienna RNA package [22]. Adjacent subsequences were overlapped by 50 nt. This method can derive RNA structures accurately and efficiently for two reasons: (1) Predicting small structures is more accurate and efficient than for large ones; (2) Structures with size less than 50 nt were folded twice as subsequences of two different larger structures, further increasing the chance of getting accurate RNA structures. We also used the setting in the Vienna package that yielded multiple RNA structures with the same minimum energy for a given sequence to further improve the folding accuracy. On the other hand, since we only obtained RNA structures derived from 100 nt subsequences of UTRs, our discovery was limited to small structures, such as short stem-loops. Thus, large RNA structures, such as IRES and SECIS, are not analyzed in this study. This step resulted in 575,410 RNA structures from human UTRs and 445,106 RNA structures from mouse UTRs.

**Figure 2:**
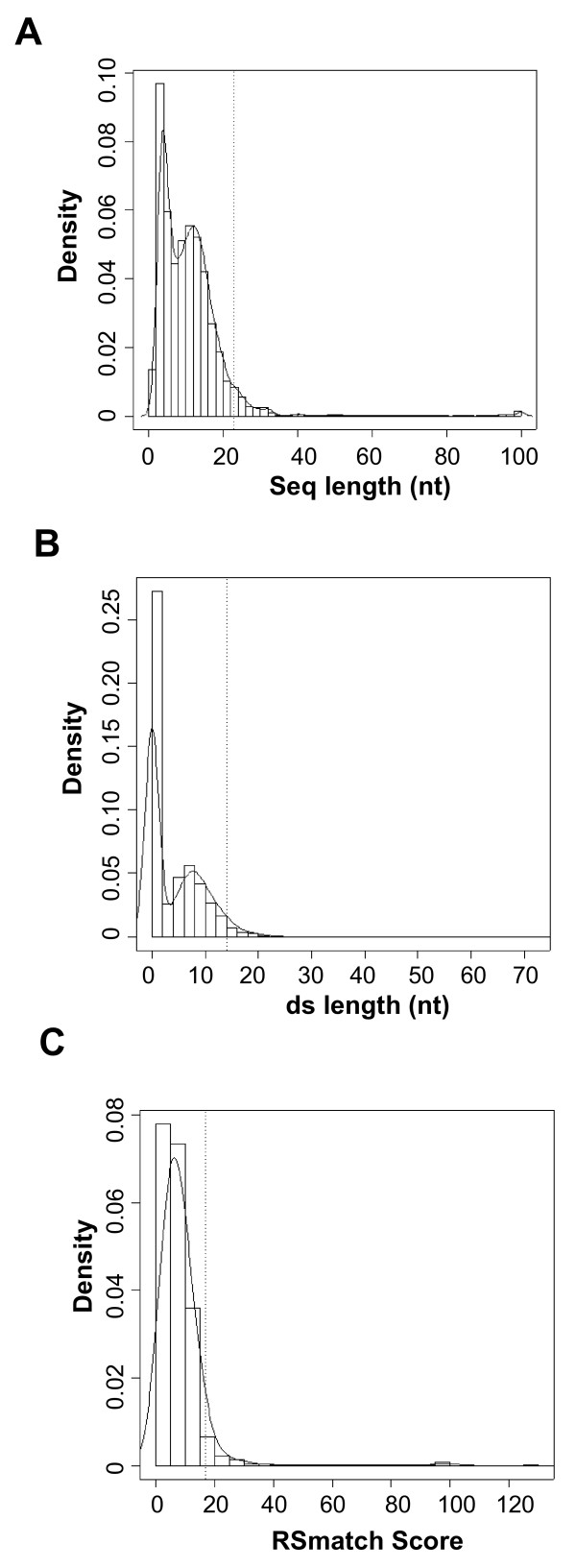
**Characteristics of aligned RNA structures in human and mouse UTRs**. Structures in human UTRs were aligned with those in mouse UTRs from orthologous genes. (A) Distribution of overall structure length. (B) Distribution of ds region length. (C) Distribution of RSmatch alignment score. Dotted vertical lines are cutoff values derived from randomized structures.

We then compared RNA structures from human and mouse orthologs (10,667 pairs in total). For each orthologous gene pair, we compared the set of RNA structures from the human gene with the set of structures from the mouse gene using RSmatch [21], which aligns RNA structures by taking into account both sequence and structure information. Alignments with a positive score from RSmatch were kept. In order to assess the significance of the alignments, we focused on three values of a structure alignment: size of the alignment, size of the double-stranded region of the alignment, and RSmatch score of the alignment. The distributions of the values for all alignments are shown in Figure 2. To select significant structures, we applied a randomization method to obtain expected values. Since most known RNA sequence elements in UTRs have the length around 6 nt, we randomized sequences by shuffling hexamers in UTRs with the goal of separating sequence conservation from structure conservation. For each aforementioned value type, the cutoff value was the 95th percentile of all values from the randomized set. They were found to be 23 nt, 14 nt, and 17 for the size of an aligned structure, the size of a ds region, and the RSmatch score, respectively. To balance selectivity and sensitivity, we retained structure alignments that had at least two of three values higher than the respective cutoff values. We eliminated structure alignments in which two matching structures had identical sequences, as we were interested in elements conserved on the structure level in this study, and it was not possible to differentiate structure conservation from sequence conservation for those alignments. We reasoned, however, that the ~100 million years since the split of human and mouse ancestors should have given functional RNA structures enough time to have random mutations in insignificant parts of the structure and compensatory mutations in the structure, and the sequences are not expected to be identical unless sequence constraint is also in play. This step resulted in 6,345 alignments.

**Figure 3:**
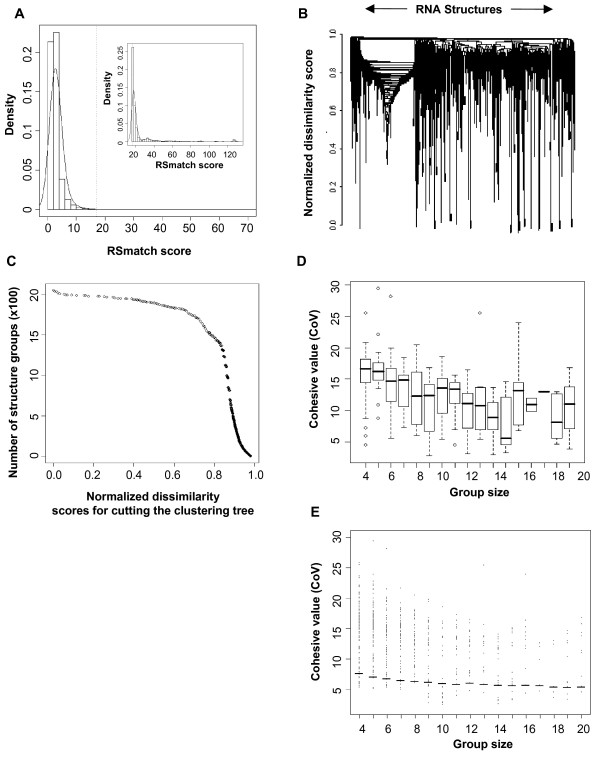
**Selection of significant RNA structure groups**. (A) Distribution of RSmatch scores for all-against-all pairwise comparisons of 6,345 human RNA structures. The cutoff value = 17, as indicated by a dotted vertical line. The distribution of scores for the selected structures (2,054 in total) is shown in the inset. (B) Hierarchical clustering of all 2,054 human RNA structures by the normalized dissimilarity score. For cluster analysis, we used the hierarchical clustering with the "average linkage" method for joining nodes. (C) One hundred normalized dissimilarity scores were used to cut the hierarchical clustering tree to obtain structure groups. Distribution of CoV vs. group size using real data (D) and randomized data (E). Horizontal lines in E are mean values for different groups, which were used as cutoff values for selecting structure groups for the real data.

We then carried out all-against-all pairwise comparisons of all 6,345 RNA structures. To make our approach computationally efficient, we focused on human RNA structures obtained from the alignments. Each comparison yielded an alignment score. We then selected structures that were similar to at least two other structures with the alignment score > 17. We obtained 2,054 RNA structures at this step (see Figure 3A for distribution of scores). Both alignments in the single-stranded (ss) and double-stranded (ds) regions can contribute to the final RSmatch score. To assess the contribution of sequence to the selection of these structures, we randomized RNA structures by swapping nucleotides in both ss and ds regions, while keeping the overall secondary structure intact. With the same selection criteria, 851 structures from the randomized set were selected. Thus, about 40% of the selected structures are primarily due to their structure information, and the remaining 60% are due to both sequence and structure information.

To group similar RNA structures together, we applied hierarchical clustering to the data. First, using pair-wise structure alignment scores, we derived normalized dissimilarity scores to represent distances among the structures (see Methods for detail). We then constructed a hierarchical tree containing all 2,054 structures based on their mutual dissimilarities (Figure 3B and Additional file 2). The hierarchical tree can be "cut" to yield sub trees that represent RNA groups. Figure 3C gives the distribution of the number of structure groups obtained by cutting the tree at every value of normalized dissimilarity score. We selected values at every percentile of this distribution to derive 100 cut heights, i.e. 1^st ^percentile, 2^nd ^percentile, etc. Using these 100 values to cut the tree, we obtained 57, 904 groups of structures, each containing several RNA structures.

Since we were interested in structures that existed in multiple genes involved in the same pathways, we examined the RNA structure groups by their GO information for the biological process category. We applied the hypergeometric test to measure the significance of association between the genes for a structure group and GO terms (see Methods for detail). A structure group was selected for further analysis if the group was significantly associated with a GO term (p-value < 0.05), and there were at least two genes in the group that were annotated with the significant GO term. To measure how member structures in each selected group are similar to one another, we used a measurement called Cohesive Value (CoV), which was the average of all pairwise similarity scores among structures in the same group. Figure 3D shows the distribution of CoVs against group size for all groups. To assess the significance of the CoVs, we randomly selected the same number of structures from 2,054 structures to form groups and calculated their CoV values. For a given group size, we repeated the process 100 times and used the mean value as the expected CoV for groups of the same size. Since the numbers of structures in a group ranged from 4 to 20, we derived expected values for groups with 4–20 structures (Figure 3E). Groups which had a CoV below the expected values were eliminated. After GO analysis and CoV filtering, we obtained 214 structure groups, corresponding to 1,125 distinct structures.

Since one structure may exist in several groups due to the 100 height values used in cutting the hierarchical tree, we eliminated groups that overlapped with other groups with a greater number of structures and lower p-values for the associated GO terms while giving preference to groups that were highly conserved between human and mouse based on a cross-validation method described in Methods. This left us with 90 structure groups in all, corresponding to 748 distinct structures from 698 genes. Of the structures, 74 are from 5' UTRs and 674 are from the 3' UTRs. Of the groups, 58 groups contain only 3' UTR structures, 30 groups contain structures from both 5' and 3' UTR and 2 groups contain only 5' UTR structures. The top 10 groups based on CoV are shown in Table 1.

**Table 1:** Top 10 structures from the "highly conserved set" based on structure conservation.

**Group ID^1 ^(CoV^2^)**	**Structure^3^**		
**GO Entries^4^**			

**3**(HSL3) (28.13)	NM_005321:721-785 NM_021062:401–431 NM_005319:704–732 NM_003526:412–438 NM_002105:545–578 NM_003516:510–534 #=GC SS_cons	AACC-C-AAAGGCTCTTTTCAGAGCCACCCA AACC-C-AAAGGCTCTTTTCAGAGCCACCTA AACC-CAAAAGGCTCTTTTCAGAGCCACC-A --CC-C-AAAGGCTCTTTTAAGAGCCACCCA A-CCAC-AAAGGCCCTTTTAAGGGCCACC-A -A-----AAAGGCTCTTTTCAGAGCCACCCA ..........((((((....)))))).....	HIST1H1E: histone cluster 1, H1e HIST1H2BB: histone cluster 1, H2bb HIST1H1C: histone cluster 1, H1c HIST1H2BC: histone cluster 1, H2bc H2AFX: H2A histone family, member X HIST2H2AA3: histone cluster 2, H2aa3

GO:0006334 (0) nucleosome assembly GO:0007001 (0) chromosome organization and biogenesis (sensu Eukarya).			

**9**(IRE) (19.93)	NM_003234:3430–3460 NM_014585:197–237 NM_003234:3884–3912 NM_003234:3481–3509 NM_000032:13–36 NM_000146:20–40 #=GC SS_cons	TTTATCAGTGACAGAGTTCACTATAAA AACTTCAGCTACAGTGTTAGCTAAGTT ATTATCGGGAGCAGTGTCTTCCATAAT ATTATCGGAAGCAGTGCCTTCCATAAT GT--TCGTCCTCAGTGCAGGGCA--AC TG---CTTCAACAGTGTTTGGA---CG (((((.(((((......))))))))))	TFRC: transferrin receptor (p90, CD71) SLC40A1: solute carrier family 40 (iron-regulated... TFRC: transferrin receptor (p90, CD71) TFRC: transferrin receptor (p90, CD71) ALAS2: aminolevulinate, delta-, synthase 2 (side... FTL: ferritin, light polypeptide

GO:0006826 (0) iron ion transport GO:0006879 (0) iron ion homeostasis			

15 (17.40)	NM_015556:203–223 NM_018947:5349–5370 NM_000617:2349–2372 NM_018970:469–543 NM_173494:843–866 #=GC SS_cons	TCATTTAACCTTTTAAATGA AAATTTAACATTTTAAATTT TAATTTCTCAGTGGAAGTTA TATATTTTCAGTAAAATGTA TATTGTGACCATTTACAGTA ((((((((....))))))))	SIPA1L1: signal-induced proliferation-associated 1 like... CYCS: cytochrome c, somatic, nuclear gene encoding SLC11A2: solute carrier family 11 (proton-coupled dival... GPR85: G protein-coupled receptor 85 CXorf41: chromosome X open reading frame 41

GO:0006810 (0.012265) transport			

17 (17.33)	NM_004441:3717–3813 NM_004443:3616–3640 NM_005398:2077–2107 NM_032827:2394–2416 #=GC SS_cons	TCTTCATATTGAAGA TCTTCATATTGAAGA CCTTCATATTGAAGG GCTTCAAATTGAAGT (((((.....)))))	EPHB1: EPH receptor B1 EPHB3: EPH receptor B3 PPP1R3C: protein phosphatase 1, regulatory (inhibitor) subu... ATOH8: atonal homolog 8 (Drosophila)

GO:0007169 (0.00033) transmembrane receptor protein tyrosine kinase signaling pathway GO:0007165 (0.031793) signal transduction GO:0006468 (0.00927) protein amino acid phosphorylation			

19 (17.17)	NM_000314:502–530 NM_032564:144–170 NM_014751:110–164 NM_016233:2056–2074 #=GC SS_cons	CCTCCCGCTCCTGGAGCGGGGGG GCCCTGGCCCCGGGGGCCGGGGC -CGCTGGC-CCCGG-GTCAGCG- -CCTGTCC-CCCTG-GGGCGGG- ((((((((((...))))))))))	PTEN: phosphatase and tensin homolog (mutated in multi... DGAT2: diacylglycerol O-acyltransferase homolog 2 (mou... MTSS1: metastasis suppressor 1 PADI3: peptidyl arginine deiminase, type III

GO:0045786 (0.00108) negative regulation of cell cycle GO:0007049 (0.009836) cell cycle GO:0006629 (0.001806) lipid metabolism			

21 (17.00)	NM_000899:1060–1087 NM_015355:3606–3643 NM_003081:1331–1430 NM_002893:1613–1645 #=GC SS_cons	TTGCTTCATAAATGAAGCAG ATTCTTTATTTATAAAGGAT -TTATGCATTTATGCATGA- --GCTTGATTTATCAAGC-- ((((((((....))))))))	KITLG: KIT ligand SUZ12: suppressor of zeste 12 homolog (Drosophila) SNAP25: synaptosomal-associated protein, 25 kDa RBBP7: retinoblastoma binding protein 7

GO:0016568 (0.000785) chromatin modification GO:0008283 (0.002712) cell proliferation			

23 (16.90)	NM_001546:1287–1309 NM_020834:2941–2962 NM_005643:1316–1339 NM_017617:8938–8965 NM_016120:2737–2778 #=GC SS_cons	CATCTATTGTTTAAAATAGATG CAGGTTTGGTTTTACAAACCTG CTTTAATGGTTTCACATTGAAG G-GATTTTGTTTAAAAAATC-T C--ATTT-GTTTAA-AAAT--G ((((((((......))))))))	ID4: inhibitor of DNA binding 4, dominant negative ... KIAA1443: KIAA1443 TAF11: TAF11 RNA polymerase II, TATA box binding pr... NOTCH1: Notch homolog 1, translocation-associated (... RNF12: ring finger protein 12

GO:0016568 (0.000785) chromatin modification GO:0008283 (0.002712) cell proliferation			

25 (16.80)	NM_004625:1678–1700 NM_015508:3801–3828 NM_031371:4664–4722 NM_016513:2522–2555 NM_004744:2276–2294 NM_138290:1864–1884 #=GC SS_cons	ATATTAATTTATTTAATTAAA ATATTTATTTTTTTAATAAAA ATATTAAAGATTCTCTTTAAA ---TTAAAGTTTTTTTTTAA- ---TTAATTTTTCAAATTAA- ---GTAAATGTTTAATTTAC- ...((((((.....)))))).	WNT7A: wingless-type MMTV integration site family, me... TIPARP: TCDD-inducible poly(ADP-ribose) polymerase ARID4B: AT rich interactive domain 4B (RBP1-like), tr... ICK: intestinal cell (MAK-like) kinase, transcript va... LRAT: lecithin retinol acyltransferase (phosphatidylc... RPIB9: Rap2-binding protein 9

GO:0007275 (0.036763) development			

27 (16.57)	NM_000252:3053–3080 NM_003582:2041–2075 NM_001635:2828–2849 NM_001338:2060–2081 NM_152267:3108–3127 NM_006329:2418–2435 NM_005627:1871–1929 NM_000170:3730–3747 #=GC SS_cons	TTTTACAATGATTTGTAAAG TTTTTATATGATTATAAAAG GTTTTGCCTAATGGCAAAAC ATTTTTCTTATTAGAAAAAT ATTTTCACTGTTGTGAAAGT -TTTTGAGTATTTTTAAAA- -TCTTCCATATTTGGAAGA- -TTATTAGTATTCTAATAA- ((((((((....))))))))	MTM1: myotubular myopathy 1 DYRK3: dual-specificity tyrosine-(Y)-phosphorylation r... AMPH: amphiphysin (Stiff-Man syndrome with breast canc... CXADR: coxsackie virus and adenovirus receptor FLJ38628: hypothetical protein FLJ38628 FBLN5: fibulin 5 SGK: serum/glucocorticoid regulated kinase GLDC: glycine dehydrogenase (decarboxylating; glycine ...

GO:0007155 (0.027609) cell adhesion			

29 (16.30)	NM_002025:8958–8980 NM_014506:1434–1458 NM_014417:1285–1350 NM_007011:2104–2126 NM_004215:1327–1350 #=GC SS_cons	GCTGATGCTTTCAGC GCTGTTCTTTGCAGC -CTCCTCCTGGGAG- -CTCTTCCTGGGAG- -CTAGTGTTTCTAG- (((((.....)))))	AFF2: AF4/FMR2 family, member 2 TOR1B: torsin family 1, member B (torsin B) BBC3: BCL2 binding component 3 ABHD2: abhydrolase domain containing 2 EBAG9: estrogen receptor binding site associated, antigen, 9...

GO:0006915 (0.011186) apoptosis			

^1 ^Group ID is a serial number, which can be used to query the GLEAN-UTR database.

^2^CoV, cohesive value, which reflects the conservation of structure.

^3^Structures are aligned, and a consensus structure is shown for each group. For each structure, its location in RefSeq sequence is indicated, and its gene symbol and name are also listed.

^4^Significant GO terms associated with each structure group are shown and p-values from hypergeometric tests are indicated in parenthesis.

HSL3 and IRE are ranked among the top hits with respect to CoV values (1^st ^and 2^nd^) as can be seen in Table 1. This result not only validated our approach, but also indicated that other groups of RNA structures may also exist, though probably not as well conserved as HSL3 or IRE. Using the multiple alignment function of RSmatch, we generated a consensus structure for each structure group. In a sense, each structure group represents a putative RNA structure element type. The sizes of the consensus structures ranged from 15 to 31. All groups and structures can be downloaded from our lab web site as a batch file [23], or searched, retrieved and viewed through an on-line database named GLEAN-UTR DB [24].

To assess the FDR for our method, we repeated all steps using randomized human and mouse UTR sequences maintaining overall dimer frequencies, and calculated the number of selected entries at each step (Additional file 3). At the last step, this randomized set resulted in 17 groups consisting of 110 human structures. Thus, the FDR is ~18.89% for the groups and ~14.71% for the structures. Of these groups, 3 groups with 14 structures also passed the cross-validation with mouse orthologs, giving FDR ~8.82% for the groups and ~5.96% for the structures.

### Comparison with other genome-wide RNA structure studies

**Figure 4:**
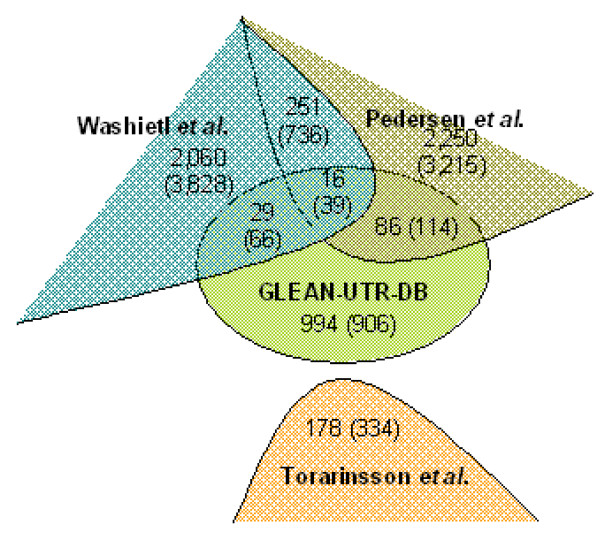
**Comparison of results from 4 RNA structure mining studies**. The Venn diagram shows overlapping structures in UTRs among the results reported by Washietl et al., Pedersen et al., Torarinsson et al., and in this study. The number in the parenthesis indicates the number of overlapped structures if only genomic regions are considered, i.e. without consideration of strand.

We next wanted to examine how the structures that we found in this study differed from and overlapped with the results obtained in other studies that have been recently carried out for finding conserved RNA structure regions in the human genome [18-20]. Using 8-way human-referenced vertebrate genome alignments, Washietl et al. detected 91,676 conserved RNA structures (at P > 0.5) using the RNAz program, which identifies RNA structures with similar thermodynamic stabilities across species. Pederson et al. developed phylogenetics stochastic context-free grammar (phylo-SCFG), and identified 48,479 candidate RNA structures using the same genome alignments. Torarinsson et al. focused on human and mouse genomic sequences that could not be aligned on the sequence level, and identified conserved structures by FOLDALIGN, a tool that simultaneously predicts and aligns RNA structures. We first identified all the structures reported by these studies that are located in UTRs, and compared them with structures found in this study. Of the 1,125 structures that were identified prior to removal of redundant groups (see above), we found 131 (12%) structures overlapped with those reported by Washietl et al and Pedersen et. al. (Figure 4 and Additional file 4). If only the genomic region is examined (without consideration of the strand), 219 (19%) structures were found to be overlapping with those in these two studies. Of the 178 structures predicted by Torarinsson et al. that overlapped with UTR regions, none of them appeared in our final result. A detailed analysis found that this was caused by differences in human and mouse UTR coverage (127 cases), gene ortholog information (27 cases), or structure alignment (24 cases).

## Discussion

We have designed a systematic approach to identify RNA structure elements conserved in human and mouse UTRs which may function coordinately in post-transcriptional regulation of biological pathways. This approach contains three major steps: (1) compare RNA structures between orthologous genes; (2) compare RNA structures among all genes; and (3) select RNA structure groups significantly associated with certain GO terms. Presumably, mRNAs containing RNA structure elements from the same group can be coordinately regulated via *trans*-acting protein factors, like those having HSL3 and IRE, leading to concerted modulation of a biological pathway. We applied this method to mining small RNA structures in this study, primarily because those structures can be more accurately predicted by RNA prediction programs using only thermodynamic parameters. As more powerful RNA structure prediction programs become available, particularly those reliant on phylogenetic information for structure prediction, this approach can be extended to larger RNA structures. The major strength of our approach is the ability to assign functions to candidate RNA structures in the genome. In addition, it may help improve the accuracy in RNA structure identification, as structures shared by multiple genes can be more reliable than those encoded by a single gene.

The assessment of FDR is critical in RNA structure analysis [25]. Using randomized sequences, we estimated a FDR of 15% for the structures identified in this study. False negative rate or sensitivity is another important issue, particularly in this study in which stringent cutoff values were applied at multiple steps. However, it is difficult to address due to lack of knowledge on true positive structure groups. We examined two well-known RNA structure elements, HSL3 and IRE, for sensitivity. For HSL3 and IRE genes that have orthologous gene information, we found that 35% (6 out of 17) HSL3 elements and 60% (6 out of 9) IRE elements are included in our final result. Thus the sensitivity can be low for some structure groups and high for others. Several steps can result in exclusion of conserved functional RNA structures in our method. First, the current coverage of orthologous genes and UTRs is not complete. In fact, most of the human HSL3 true positive structures (44 in total) were not even analyzed in this study due to lack of orthologous gene or UTR information. This will improve as more comprehensive gene annotations, and more accurate transcription start sites and polyadenylation sites are available. Second, it is known that RNA structure prediction by thermodynamic parameters has limitation in accuracy [26]. Third, some structures may reside in genes for which GO information is not adequately annotated.

One potential approach to improve sensitivity is to search the genome with consensus RNA structures derived from the groups. We tested this idea by first generating RNA structure patterns for the groups and used them to search human UTRs by PatSearch [27]. Candidate elements were further analyzed for GO terms to ensure consistency in their association with biological pathways as the original groups. As expected, the group size increased exponentially (Additional file 5). While this approach seems promising in reducing the false negative rate, the control for false positive rate needs to be further developed. We leave this work for future exploration.

About 12% of the structures identified in this work overlap those reported in other studies (Figure 4). Interestingly, each genome-wide approach resulted in a large fraction of unique structures, suggesting that RNA structure identification is largely influenced by the chosen method. Many structures in UTRs identified by other studies are not in our final result (Figure 4). This is attributable to several aspects of the design of our study, in addition to the technical difference and false negative issues described above. First, our analysis is based on RNA structure groups, and functional structures located in individual genes are not included. We found this is the case for several recently reported RNA structures in UTRs [28,29]. Second, RNA structures with similar functions but different secondary structures, like IRES, cannot be identified. Third, large structures, like SECIS, are not examined. Notwithstanding these issues, the structures that overlap between this study and others are of higher importance for further wet lab validations (Additional file 4).

In summary, our result indicates that there may exist many conserved stem-loop structures in human UTRs that are involved in coordinate post-transcriptional gene regulation of biological pathways, similar to HSL3 and IRE structures. This bioinformatics study lays a ground work for future wet lab validations of putative RNA stem-loop groups and represents a framework which can be used to analyze RNA structures identified by other approaches and in other species.

## Methods

### UTR sequence and structure databases

We downloaded 28,926 human and 26,243 mouse RefSeq mRNA sequences from NCBI. UTRs of RefSeq sequences were extracted according to RefSeq's GenBank annotation. The information regarding human and mouse orthologs was obtained from the HomoloGene database. We prepared RNA structures in UTRs by a method called "slide and fold" as described previously [21]. Briefly, for each UTR sequence, we took 100 nt subsequences at every 50 nt nucleotide position from 5' to 3' resulting in consecutive subsequences overlapping with one another on a 50 nt segment. Subsequences shorter than 100 nt, e.g. at the 5' or 3' ends, were also kept. We then folded all of the subsequences using the RNAsubopt function in the Vienna RNA package [22], with the setting "-e 0". With this setting, multiple structures with the same minimum energy can be generated. Using this method, we obtained 575,410 structures from human UTRs, and 445,106 structures from mouse UTRs.

### RNA structure comparison

Pairwise comparisons of RNA structures (human vs. mouse and human vs. human) were carried out by RSmatch [21], with the "dsearch" function and default scoring matrices for ss and ds regions. Specifically, nucleotide match scores were 1 and 3 in single-stranded (ss) and double-stranded (ds) regions, respectively; and mismatch scores were -1 and 1, in ss and ds regions, respectively. Gap penalty was -6 for both ss and ds regions. This scoring scheme in effect gave more weight on matches in ds regions than those in ss regions. Randomization of RNA structure was carried out by a PERL script.

### Cluster analysis of RNA structures

To cluster RNA structures, we calculated normalized dissimilarity scores D_i, j _between all structures: D_i, j _= (S_max_-S_i,j_)/S_max_, where S_i, j _was the similarity score derived from RSmatch using the local structure alignment function between structures i and j, and S_max _was the maximum similarity score obtained from all structure comparisons. For cluster analysis, we used the hierarchical clustering function in R [30], with the "average linkage" method for joining nodes. To select groups of RNA structures, we applied the "cutree" function to cut the hierarchical tree obtained from R into groups using the normalized dissimilarity scores, which were also called heights in the tree. Structures in each group were aligned by the multiple structure alignment function of RSmatch with default scoring matrices. Structures in the same group were also compared in a pairwise manner; the average of all pair-wise similarity scores for the group was called the Cohesive Value (CoV) of that group, which indicated the degree of similarity among structures in the group.

### Gene Ontology analysis

The biological process (BP) category of Gene Ontology (GO) was downloaded from the GO database [31]. The mapping between genes and GO entries was obtained from NCBI Gene database [32]. A hypergeometric test was used to assess whether an RNA structure group was significantly associated with some GO entries. Briefly, in the hypergeometric test, there are four parameters: (1) *m*, the number of white balls in an urn, (2) *n*, the number of black balls in the urn, (3) *k*, the number of balls drawn from the urn, and (4) *x*, the number of white balls drawn from the urn. The probability that *x *out of the *k *balls drawn are white from the urn containing *m *+ *n *balls is

(1) $$f(x,m,n,k)=\frac{\left(\begin{array}{l} m\\ x \end{array}\right)\left(\begin{array}{l} n\\ k-x \end{array}\right)}{\left(\begin{array}{l} m+n\\ k \end{array}\right)}$$

For each RNA structure group *M *containing multiple genes, all GO entries are examined to evaluate their associations with *M *. Through the mapping information between *M *and a GO entry *G *in a GO category *C*, we are able to calculate four numbers: (1) *N1*, the number of genes associated with any GO entry in *C*, (2) *N2*, the number of genes associated with *G *in *C*, (3) *N3*, the number of genes in *M *associated with any GO entry in *C*, and (4) *N4*, the number of genes in *M *associated with *G *in *C*, where *N1 *≥ *N2 *and *N3 *≥ *N4*. The p-value of the GO entry *G *is calculated by *p(G) *= *f(N4, N2, N1 *– *N2, N3)*, where the function *f *is defined in equation 1.

### Cross-validation with mouse UTR structures

After performing the GO analysis and CoV filtering, we cross-validated selected human RNA structure groups with their orthologous mouse structures. For each group, mouse UTR structures corresponding to human structures in the group were retrieved. Then the mouse UTR structure which has the highest similarity to a human structure was selected. All these selected mouse structures were compared by the multiple structure alignment function of RSmatch which also gave the consensus structure. The consensus structure of human RNA structures was then compared to that of mouse one. An RNA structure group was considered to be highly conserved if: (1) the human consensus was identical to the mouse one, or (2) the human consensus was contained within the mouse one or vice verse. In case (2), a consensus of human and mouse structures was built to represent the structure group.

### Comparison with structure elements from other studies

The datasets for Pedersen et al. and for Washietl et al. were downloaded from their respective web sites [18,19]. The dataset from Torarinsson et al. was obtained from the authors. We used BLAT to find genomic locations for all structure elements, including ours, and identified overlapped ones by their locations.

## Authors' contributions

BT conceived of the study and designed the methods. JL did preliminary studies. MK extended and finished the work with help from DW. BT and MK wrote the manuscript. JTLW participated in mentoring JL, MK, and DW, and writing the manuscript. All authors read and approved the final manuscript.

## Supplementary Material

Additional file 1 **Graphical representations HSL3 (A) and IRE (B)**. The structures are also represented in the dot-bracket form. Click here for file

Additional file 2 **Heat map for all-against-all comparisons of 2,054 human RNA structures**. The normalized dissimilarity score is represented by color based on the scale shown at the bottom. The structures are in the same order as those shown in the hierarchical clustering tree in Figure 3B. Click here for file

Additional file 3 **GLEAN-UTR for randomized UTR sequences**. UTR sequences randomized by 1-order Markov chain were subject to the same GLEAN-UTR approach as shown in Figure 1. The numbers of structures and structure groups are shown in parenthesis. Click here for file

Additional file 4 Structures identified both by this study and by Washietl et al. or Pedersen et al. Click here for file

Additional file 5 **Extending RNA structure groups by PatSearch**. The 90 structure groups were used to search human UTRs to obtain additional group members using PatSearch. GO analysis refers to filtering out hits without the same GO term annotation as the original group. The structure groups are ordered according to the difference between the original group size and the group size after PatSearch. Click here for file

## References

[B1] Mignone F, Gissi C, Liuni S, Pesole G (2002). Untranslated regions of mRNAs. Genome Biol.

[B2] Wilkie GS, Dickson KS, Gray NK (2003). Regulation of mRNA translation by 5'- and 3'-UTR-binding factors. Trends Biochem Sci.

[B3] Kuersten S, Goodwin EB (2003). The power of the 3' UTR: translational control and development. Nat Rev Genet.

[B4] Keene JD, Tenenbaum SA (2002). Eukaryotic mRNPs may represent posttranscriptional operons. Mol Cell.

[B5] Bakheet T, Frevel M, Williams BR, Greer W, Khabar KS (2001). ARED: human AU-rich element-containing mRNA database reveals an unexpectedly diverse functional repertoire of encoded proteins. Nucleic Acids Res.

[B6] Wilusz CJ, Wilusz J (2004). Bringing the role of mRNA decay in the control of gene expression into focus. Trends Genet.

[B7] Bartel DP (2004). MicroRNAs: genomics, biogenesis, mechanism, and function. Cell.

[B8] Filipowicz W, Bhattacharyya SN, Sonenberg N (2008). Mechanisms of post-transcriptional regulation by microRNAs: are the answers in sight?. Nat Rev Genet.

[B9] Baird SD, Turcotte M, Korneluk RG, Holcik M (2006). Searching for IRES. Rna.

[B10] Rouault TA (2006). The role of iron regulatory proteins in mammalian iron homeostasis and disease. Nat Chem Biol.

[B11] Grundner-Culemann E, Martin GW, Harney JW, Berry MJ (1999). Two distinct SECIS structures capable of directing selenocysteine incorporation in eukaryotes. RNA.

[B12] Marzluff WF (2005). Metazoan replication-dependent histone mRNAs: a distinct set of RNA polymerase II transcripts. Curr Opin Cell Biol.

[B13] Hu J, Lutz CS, Wilusz J, Tian B (2005). Bioinformatic identification of candidate cis-regulatory elements involved in human mRNA polyadenylation. RNA.

[B14] Rajewsky N (2006). microRNA target predictions in animals. Nat Genet.

[B15] Matlin AJ, Clark F, Smith CWJ (2005). Understanding alternative splicing: towards a cellular code. Nat Rev Mol Cell Biol.

[B16] Ladd AN, Cooper TA (2002). Finding signals that regulate alternative splicing in the post-genomic era. Genome Biol.

[B17] John B, Sander C, Marks DS (2006). Prediction of human microRNA targets. Methods Mol Biol.

[B18] Washietl S, Hofacker IL, Lukasser M, Huttenhofer A, Stadler PF (2005). Mapping of conserved RNA secondary structures predicts thousands of functional noncoding RNAs in the human genome. Nat Biotechnol.

[B19] Pedersen JS, Bejerano G, Siepel A, Rosenbloom K, Lindblad-Toh K, Lander ES, Kent J, Miller W, Haussler D (2006). Identification and classification of conserved RNA secondary structures in the human genome. PLoS Comput Biol.

[B20] Torarinsson E, Sawera M, Havgaard JH, Fredholm M, Gorodkin J (2006). Thousands of corresponding human and mouse genomic regions unalignable in primary sequence contain common RNA structure. Genome Res.

[B21] Liu J, Wang JT, Hu J, Tian B (2005). A method for aligning RNA secondary structures and its application to RNA motif detection. BMC Bioinformatics.

[B22] Hofacker IL (2003). Vienna RNA secondary structure server. Nucleic Acids Research.

[B23] GLEAN-UTR text files. http://exon.umdnj.edu/GLEAN-UTR.

[B24] GLEAN-UTR-DB. http://datalab.njit.edu/biodata/GLEAN-UTR-DB/.

[B25] Babak T, Blencowe BJ, Hughes TR (2007). Considerations in the identification of functional RNA structural elements in genomic alignments. BMC Bioinformatics.

[B26] Mathews DH, Sabina J, Zuker M, Turner DH (1999). Expanded sequence dependence of thermodynamic parameters improves prediction of RNA secondary structure. Journal of Molecular Biology.

[B27] Grillo G, Licciulli F, Liuni S, Sbisa E, Pesole G PatSearch: a program for the detection of patterns and structural motifs in nucleotide sequences. Nucleic Acids Research.

[B28] Sarnowska E, Grzybowska EA, Sobczak K, Konopinski R, Wilczynska A, Szwarc M, Sarnowski TJ, Krzyzosiak WJ, Siedlecki JA (2007). Hairpin structure within the 3'UTR of DNA polymerase beta mRNA acts as a post-transcriptional regulatory element and interacts with Hax-1. Nucleic Acids Res.

[B29] Brenet F, Dussault N, Delfino C, Boudouresque F, Chinot O, Martin PM, Ouafik LH (2006). Identification of secondary structure in the 5'-untranslated region of the human adrenomedullin mRNA with implications for the regulation of mRNA translation. Oncogene.

[B30] Venables WN, Ripley BD, Chambers J, Eddy W, Hardle W, Sheather S, Tierney L (2002). Modern Applied Statistics with S. Statistics and Computing.

[B31] Ashburner M, Ball CA, Blake JA, Botstein D, Butler H, Cherry JM, Davis AP, Dolinski K, Dwight SS, Eppig JT, Harris MA, Hill DP, Issel-Tarver L, Kasarskis A, Lewis S, Matese JC, Richardson JE, Ringwald M, Rubin GM, Sherlock G (2000). Gene ontology: tool for the unification of biology. The Gene Ontology Consortium. Nat Genet.

[B32] Pruitt KD, Maglott DR (2001). RefSeq and LocusLink: NCBI gene-centered resources. Nucleic Acids Res.

