# Supplementary material for: Mining small RNA structure elements in untranslated regions of human and mouse mRNAs using structure-based alignment
Source: BMC Genomics. 2008 Apr 25;9:189. doi: 10.1186/1471-2164-9-189 (PMC2413145; doi:10.1186/1471-2164-9-189)
Supplement: Additional file 1 — Graphical representations HSL3 (A) and IRE (B). The structures are also represented in the dot-bracket form. [file 1471-2164-9-189-S1.pdf]

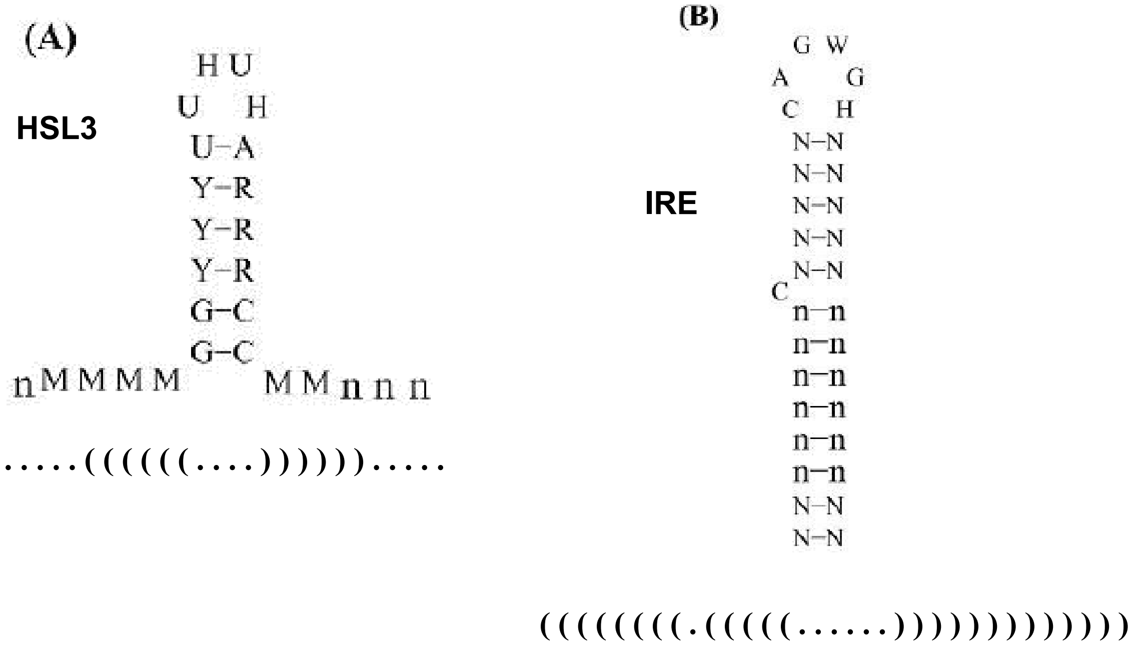

Additional file 1. The graphical representation of HSL3 motif (A) and IRE motif (B). The structures are also represented in the dot-bracket forms.
