# Supplementary material for: Mining small RNA structure elements in untranslated regions of human and mouse mRNAs using structure-based alignment
Source: BMC Genomics. 2008 Apr 25;9:189. doi: 10.1186/1471-2164-9-189 (PMC2413145; doi:10.1186/1471-2164-9-189)
Supplement: Additional file 2 — Heat map for all-against-all comparisons of 2,054 human RNA structures. The normalized dissimilarity score is represented by color based on the scale shown at the bottom. The structures are in the same order as those shown in the hierarchical clustering tree in Figure 3B. [file 1471-2164-9-189-S2.pdf]

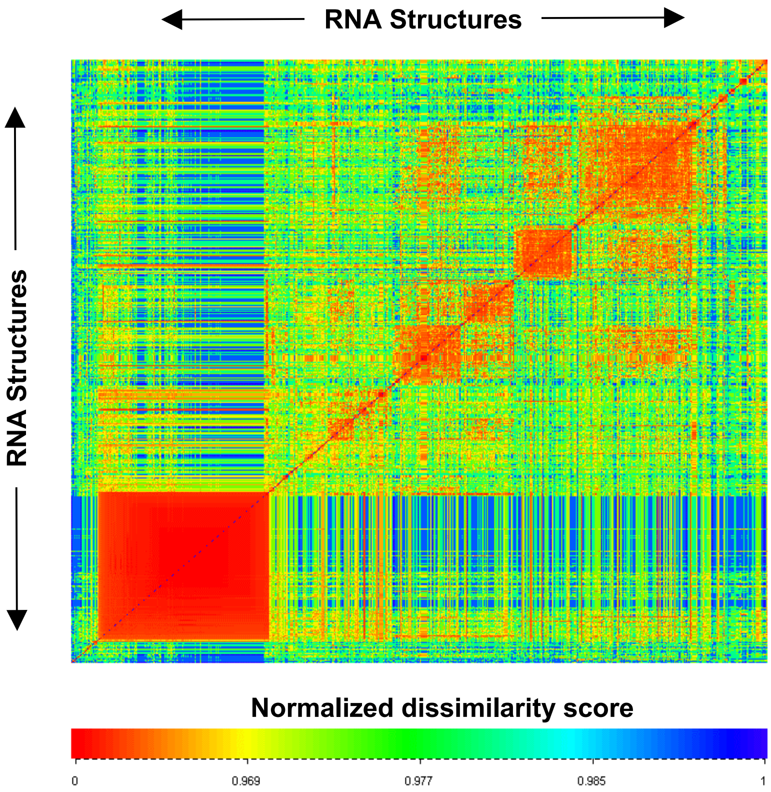

Additional file 2. Heat map for all-against-all comparisons of 2,054 human RNA structures. The normalized dissimilarity score is represented by color based on the scale shown at the bottom. The structures are in the same order as those shown in the hierarchical clustering tree in Figure 3B.
