# Supplementary material for: Mining small RNA structure elements in untranslated regions of human and mouse mRNAs using structure-based alignment
Source: BMC Genomics. 2008 Apr 25;9:189. doi: 10.1186/1471-2164-9-189 (PMC2413145; doi:10.1186/1471-2164-9-189)
Supplement: Additional file 3 — GLEAN-UTR for randomized UTR sequences. UTR sequences randomized by 1-order Markov chain were subject to the same GLEAN-UTR approach as shown in Figure 1. The numbers of structures and structure groups are shown in parenthesis. [file 1471-2164-9-189-S3.pdf]

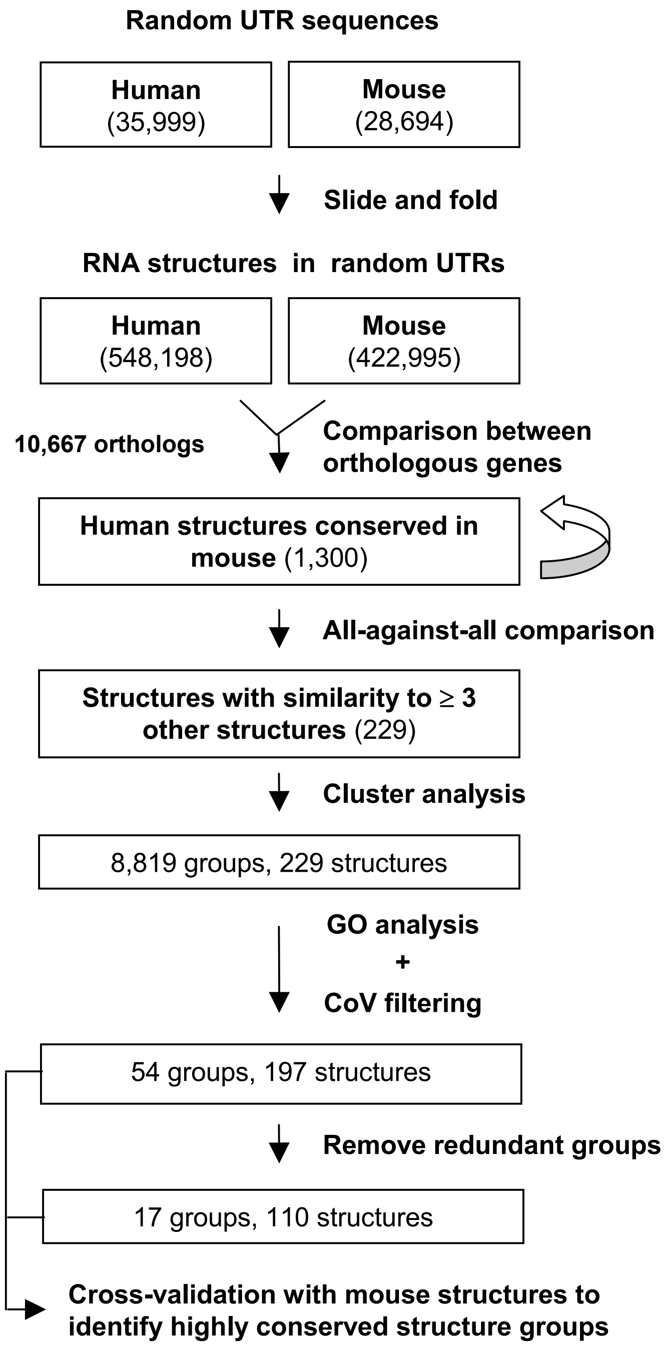

Additional file 3. GLEAN-UTR for randomized UTR sequences. UTR sequences randomized by 1-order Markov chain were subject to the same GLEAN-UTR approach as shown in Figure 1. The numbers of structures and structure groups are shown in parenthesis.
