# Supplementary material for: Mining small RNA structure elements in untranslated regions of human and mouse mRNAs using structure-based alignment
Source: BMC Genomics. 2008 Apr 25;9:189. doi: 10.1186/1471-2164-9-189 (PMC2413145; doi:10.1186/1471-2164-9-189)
Supplement: Additional file 4 — Structures identified both by this study and by Washietl et al. or Pedersen et al. [file 1471-2164-9-189-S4.pdf]

Additional file 4. Structures identified both by this study and by Washietl et al. or Pedersen et al.

Group ID[1]	RefSeq ID	Annotation	Structure	Overlap with[2]
115	NM_005922:5007-5032	Homo sapiens mitogen-activated protein kinase kinase kinase 4 (MAP3K4), transcript variant 1, mRNA	TATGTAATATTTACATA<br>(((((((...)))))))	P
156	NM_006265:3592-3636	Homo sapiens RAD21 homolog (S. pombe) (RAD21), mRNA	TATAACTTTTCTAATAAAAGTTGTG<br>((((((((((.....))))))))))	P
193	NM_007203:3560-3639	Homo sapiens A kinase (PRKA) anchor protein 2 (AKAP2), transcript variant 1, mRNA	ATTTTAATGGACTATTTATTAAAGT<br>(((((((((((...)))))))))))	P
179	NM_139168:3644-3678	Homo sapiens splicing factor, arginine/serine-rich 12 (SFRS12), mRNA	GCTTTACTATGTAAAGT<br>(((((((...)))))))	P
224	NM_018959:1740-1782	Homo sapiens DAZ associated protein 1 (DAZAP1), transcript variant 2, mRNA	TATGTTAAAGAAAAAATATA<br>((((((........))))))	P
180	NM_005249:2317-2397	Homo sapiens forkhead box G1B (FOXG1B), mRNA	TGTATATTTTGATGTATG<br>(((((((....)))))))	P, W
128	NM_173469:2838-2914	Homo sapiens hypothetical protein LOC92912 (LOC92912), mRNA	TAAACTTGCATCAAGTTTA<br>((((((((...))))))))	P
215	NM_004093:4035-4084	Homo sapiens ephrin-B2 (EFNB2), mRNA	ATTGCTGCATATTTGTGCCGTAAT<br>(((((.((((....)))).)))))	P
124	NM_020245:10602-10629	Homo sapiens tubby like protein 4 (TULP4), mRNA	TTTGCATTTGTTTATAAATGCATTATTT<br>..((((((((....))))))))......	P, W
186	NM_004396:2183-2242	Homo sapiens DEAD (Asp-Glu-Ala-Asp) box polypeptide 5 (DDX5), mRNA	CCTGAAACAATTTTTAGGT<br>(((((((....))))))).	P

11	NM_001546:1287-1309	Homo sapiens inhibitor of DNA binding 4, dominant negative helix-loop-helix protein (ID4), mRNA	CATCTATTGTTTAAAATAGATG<br>((((((((......))))))))	P, W
13	NM_005627:1871-1929	Homo sapiens serum/glucocorticoid regulated kinase (SGK), mRNA	TCTTCCATATTTGGAAGA<br>(((((((....)))))))	P
115	NM_022900:3428-3449	Homo sapiens O-acetyltransferase (CAS1), mRNA	TTTCCAATATTTGGAAA<br>(((((((...)))))))	P
159	NM_004235:2401-2458	Homo sapiens Kruppel-like factor 4 (gut) (KLF4), mRNA	TGTGCAATAATTTGTACA<br>(((((((....)))))))	P
67	NM_005204:2736-2767	Homo sapiens mitogen-activated protein kinase kinase kinase 8 (MAP3K8), mRNA	ATTCAAAACGTGATGTTTTGAAT<br>(((((((((.....)))))))))	P, W
87	NM_014795:5073-5162	Homo sapiens zinc finger homeobox 1b (ZFHX1B), mRNA	AAATAACATTTTATTT<br>((((((....))))))	P
277	NM_016131:1409-1508	Homo sapiens RAB10, member RAS oncogene family (RAB10), mRNA	TAAAGTTAGAATTAACAATTTTA<br>(((((((.(......))))))))	P
117	NM_014757:5412-5436	Homo sapiens mastermind-like 1 (Drosophila) (MAML1), mRNA	TGTAAATAAATGTTTACA<br>(((((((....)))))))	P, W
9	NM_014585:197-237	Homo sapiens solute carrier family 40 (iron-regulated transporter), member 1 (SLC40A1), mRNA	AACTTCAGCTACAGTGTTAGCTAAGTT<br>(((((.(((((......))))))))))	P
193	NM_015397:2051-2087	Homo sapiens KIAA1892 (KIAA1892), mRNA	CTCAGACTTTCTGTGAAAGTTTGGG<br>(((((((((((...)))))))))))	P
128	NM_001905:2511-2531	Homo sapiens CTP synthase (CTPS), mRNA	ACTCCTTGCATCAAGGGGT<br>((((((((...))))))))	P

226	NM_006471:1171-1202	Homo sapiens myosin regulatory light chain MRCL3 (MRCL3), mRNA	AGAAAGTTATTCGCTCGATTTTTT<br>((((((((........))))))))	P
248	NM_003701:1654-1706	Homo sapiens tumor necrosis factor (ligand) superfamily, member 11 (TNFSF11), transcript variant 1, mRNA	AAATGTCTTGCTGTTGACATAT<br>(.(((((........))))).)	P
193	NM_024045:2432-2468	Homo sapiens DEAD (Asp-Glu-Ala-Asp) box polypeptide 50 (DDX50), mRNA	GTATTTTTTTAAAAAGTAT<br>((((((((...))))))))	P
209	NM_000214:4674-4767	Homo sapiens jagged 1 (Alagille syndrome) (JAG1), mRNA	TTTGATTTATTAACTTAATAATCAA<br>.((((.((((((...))))))))))	P
200	NM_004463:4228-4250	Homo sapiens faciogenital dysplasia (Aarskog-Scott syndrome) (FGD1), mRNA	TTTTTTTTTTTTTAAGAAAAA<br>((((((((.....))))))))	P
55	NM_002973:4142-4178	Homo sapiens spinocerebellar ataxia 2 (olivopontocerebellar ataxia 2, autosomal dominant, ataxin 2) (SCA2), mRNA	TGCTTCTACCAACTGGAAGCA<br>((((((((.....))))))))	P
126	NM_182789:1643-1664	Homo sapiens poly(A) binding protein interacting protein 1 (PAIP1), transcript variant 2, mRNA	TATATAATAGTTTATTATGTA<br>(((((((((...)))))))))	P
118	NM_005776:597-666	Homo sapiens cornichon homolog (Drosophila) (CNIH), mRNA	TTTAAAAAATGACTCCTTATTTTTTAAA<br>((((((((((((....))))))))))))	P
244	NM_182700:2993-3066	Homo sapiens Sp8 transcription factor (SP8), transcript variant 1, mRNA	TGTATAGTATTTTTCTTGTACA<br>(((((((........)))))))	P
186	NM_001292:1621-1646	Homo sapiens CDC-like kinase 3 (CLK3), transcript variant phclk3/152, mRNA	TGTTATAAAGTTATAATA<br>(((((((....)))))))	P
83	NM_172316:2894-2949	Homo sapiens Meis1, myeloid ecotropic viral integration site 1 homolog 2 (mouse)	TATCAGATCTGCTGTGGAATTGGTA<br>((((((.(((.....))).))))))	P

		(MEIS2), transcript variant h, mRNA		
61	NM_014497:6409-6441	Homo sapiens NP220 nuclear protein (NP220), mRNA	GGTTTGATTTTTATATCAAATC<br>((((((((......))))))))	P
85	NM_004089:656-686	Homo sapiens delta sleep inducing peptide, immunoreactor (DSIPI), transcript variant 2, mRNA	TCTGTCCTCAGGGTGGGCAGA<br>(((((((.......)))))))	P
209	NM_001827:387-441	Homo sapiens CDC28 protein kinase regulatory subunit 2 (CKS2), mRNA	GTATTCAGTGAATAC<br>((((((...))))))	P, W
109	NM_020432:3107-3145	Homo sapiens putative homeodomain transcription factor 2 (PHTF2), mRNA	GCACAGCTCCAACTGTGC<br>((((((......))))))	P, W
150	NM_015024:3682-3707	Homo sapiens exportin 7 (XPO7), mRNA	TGTATAAACATGTACA<br>((((((....))))))	P
139	NM_018287:3611-3649	Homo sapiens Rho GTPase activating protein 12 (ARHGAP12), mRNA	TATATAATTGTGTTGTATAG<br>((((((((...)))))))).	P
180	NM_013352:3919-3946	Homo sapiens squamous cell carcinoma antigen recognized by T cells 2 (SART2), mRNA	TTTATTTTCCTAAATAAA<br>(((((((....)))))))	P
9	NM_003234:3481-3509	Homo sapiens transferrin receptor (p90, CD71) (TFRC), mRNA	ATTATCGGAAGCAGTGCCTTCCATAAT<br>(((((.(((((......))))))))))	P
224	NM_005487:4142-4194	Homo sapiens high-mobility group protein 2-like 1 (HMG2L1), mRNA	GTATAAAGAAATAAAATTTTGTAC<br>((((((((........))))))))	P
126	NM_170731:1145-1229	Homo sapiens brain-derived neurotrophic factor (BDNF), transcript variant 3, mRNA	TGTATAAATGAAGTTTATACA<br>(((((((((...)))))))))	P
17	NM_004441:3717-3813	Homo sapiens EphB1 (EPHB1), mRNA	TCTTCATATTGAAGA<br>(((((.....)))))	P, W

13	NM_001635:2828-2849	Homo sapiens amphiphysin (Stiff-Man syndrome with breast cancer 128kDa autoantigen) (AMPH), transcript variant 1, mRNA	GTTTTGCCTAATGGCAAAAC<br>((((((((....))))))))	P
266	NM_002207:3233-3256	Homo sapiens integrin, alpha 9 (ITGA9), mRNA	AAAAATCTTCTCCAGATTTTT<br>((((((((.....))))))))	P
117	NM_005153:2858-2934	Homo sapiens ubiquitin specific protease 10 (USP10), mRNA	TAAAAAAGAAATTTTTTA<br>(((((((....)))))))	P
157	NM_005985:1632-1657	Homo sapiens snail homolog 1 (Drosophila) (SNAI1), mRNA	TTTGTATAGTTATATGTACAGTT<br>.(((((((......)))))))..	P
9	NM_003234:3430-3460	Homo sapiens transferrin receptor (p90, CD71) (TFRC), mRNA	TTTATCAGTGACAGAGTTCACTATAAA<br>(((((.(((((......))))))))))	P
131	NM_002971:3093-3115	Homo sapiens special AT-rich sequence binding protein 1 (binds to nuclear matrix/scaffold-associating DNA's) (SATB1), mRNA	TTGTTTTTATTTTGGAAATAA<br>((((((((.....))))))))	P
115	NM_172220:1179-1200	Homo sapiens colony stimulating factor 3 (granulocyte) (CSF3), transcript variant 3, mRNA	TGTTTAATATTTAAACA<br>(((((((...)))))))	P, W
87	NM_000110:3616-3635	Homo sapiens dihydropyrimidine dehydrogenase (DPYD), mRNA	AAAATTACCTTTAATTTT<br>(((((((....)))))))	P
128	NM_004098:2788-2879	Homo sapiens empty spiracles homolog 2 (Drosophila) (EMX2), mRNA	TATACTTCCAAGAAGTATG<br>((((((((...))))))))	P
151	NM_022138:1582-1605	Homo sapiens SPARC related modular calcium binding 2 (SMOC2), mRNA	ACATACAATGTATGT<br>((((((...))))))	P

186	NM_000266:138-190	Homo sapiens Norrie disease (pseudoglioma) (NDP), mRNA	TCTCAGAAAAGTCTGAGA<br>(((((((....)))))))	P
41	NM_015032:5209-5257	Homo sapiens androgen-induced proliferation inhibitor (APRIN), mRNA	TTTAAAGTATTTTAATTTTAAA<br>((((((((......))))))))	P
198	NM_004308:3310-3336	Homo sapiens Rho GTPase activating protein 1 (ARHGAP1), mRNA	TTTTTGTATTTCAATAAAAA<br>(((((((......)))))))	P
136	NM_003564:693-742	Homo sapiens transgelin 2 (TAGLN2), mRNA	AATATATATGTAGATATATATT<br>(((((((((....)))))))))	P
222	NM_000304:839-869	Homo sapiens peripheral myelin protein 22 (PMP22), transcript variant 1, mRNA	TTGAAGATGTATATAATATCTCCGG<br>(((.((((((.....)))))).)))	P
246	NM_025213:8264-8332	Homo sapiens spectrin, beta, non-erythrocytic 4 (SPTBN4), mRNA	GGAGGGGACACCCCCTCC<br>(((((((....)))))))	P, W
73	NM_181552:4943-4969	Homo sapiens cut-like 1, CCAAT displacement protein (Drosophila) (CUTL1), transcript variant 1, mRNA	TTTTCAAGGAAGAAAA<br>(((((......)))))	P
117	NM_148976:1421-1445	Homo sapiens proteasome (prosome, macropain) subunit, alpha type, 1 (PSMA1), transcript variant 1, mRNA	GTGAAATAAAATTGTTTCAC<br>((((((((....))))))))	P
232	NM_001149:4428-4451	Homo sapiens ankyrin 3, node of Ranvier (ankyrin G) (ANK3), transcript variant 2, mRNA	CAGAATACTAATATTTTG<br>(((((((....)))))))	P
127	NM_002268:3309-3369	Homo sapiens karyopherin alpha 4 (importin alpha 3) (KPNA4), mRNA	TTGCATAAAAGTTATGCAA<br>((((((((...))))))))	P
181	NM_005281:1740-1824	Homo sapiens G protein-coupled receptor 3 (GPR3), mRNA	TGGTTTTTTATTTTTTTAAGAAGCCA<br>((((((((((......))))))))))	P, W

148	NM_025263:2056-2088	Homo sapiens proline-rich polypeptide 3 (PRR3), mRNA	TTGTTTTCTGTGAAAACAG<br>((((((((...))))))))	P, W
85	NM_020360:1706-1732	Homo sapiens phospholipid scramblase 3 (PLSCR3), mRNA	TTTATATG--T-TACATT-CATATAAA<br>(((((((((.........)))))))))	P
193	NM_182898:2027-2094	Homo sapiens cAMP responsive element binding protein 5 (CREB5), mRNA	CATTTTTATAGTTATTATGGAAATG<br>(((((((((((...)))))))))))	P
145	NM_173560:3263-3295	Homo sapiens regulatory factor X domain containing 1 (RFXDC1), mRNA	TTATTTGACAAAAAGTCAAATGTG<br>.((((((((.....))))))))..	P
9	NM_003234:3884-3912	Homo sapiens transferrin receptor (p90, CD71) (TFRC), mRNA	ATTATCGGGAGCAGTGTCTTCCATAAT<br>(((((.(((((......))))))))))	P
214	NM_004593:1186-1279	Homo sapiens splicing factor, arginine/serine-rich 10 (transformer 2 homolog, Drosophila) (SFRS10), mRNA	AAAAGTATGTTTTGCATGTATTTTTTT<br>((((((((((.....))))))))))..	P
148	NM_002293:7875-7908	Homo sapiens laminin, gamma 1 (formerly LAMB2) (LAMC1), mRNA	ATTTTATTTATAATAAAAT<br>((((((((...))))))))	P
85	NM_198077:880-905	Homo sapiens gm117 (gm117), mRNA	ATATATTTTTAAAGTAAATATATT<br>(((((((((.......)))))))))	P
53	NM_182697:951-1050	Homo sapiens ubiquitin-conjugating enzyme E2H (UBC8 homolog, yeast) (UBE2H), transcript variant 2, mRNA	ATATATATATATTATATAT<br>(((((((.....)))))))	P
214	NM_018948:2571-2593	Homo sapiens mitogen-inducible gene 6 (MIG-6), mRNA	CAACACAAGCTGGCCTTGTGTTG<br>(((((((((.....)))))))))	P
131	NM_032291:3259-3291	Homo sapiens hypothetical protein DKFZp761D221 (DKFZp761D221), mRNA	TTTTTATAACTTGTGTAAAAA<br>((((((((.....))))))))	P

170	NM_015461:4825-4865	Homo sapiens early hematopoietic zinc finger (EHZF), mRNA	GTTTTCCAAGAGGAAAT<br>((((((.....))))))	P
162	NM_004730:1700-1796	Homo sapiens eukaryotic translation termination factor 1 (ETF1), mRNA	TGAAAAAAAATGATTTTTTTAA<br>((((((((.....)))))))).	P
85	NM_019028:2342-2383	Homo sapiens HIP14-related protein (HIP14L), mRNA	TAAATATGTAAAAAATATTTA<br>(((((((.......)))))))	P
99	NM_002167:1143-1169	Homo sapiens inhibitor of DNA binding 3, dominant negative helix-loop-helix protein (ID3), mRNA	ACAGGAAGGTGACTTTCTGT<br>((((((((....))))))))	P
85	NM_182763:1617-1650	Homo sapiens myeloid cell leukemia sequence 1 (BCL2-related) (MCL1), transcript variant 2, mRNA	TGTAAAAAT-TGTATA-TATTTTTACA<br>(((((((((.........)))))))))	P
21	NM_003081:1331-1430	Homo sapiens synaptosomal-associated protein, 25kDa (SNAP25), transcript variant 1, mRNA	TTATGCATTTATGCATGA<br>(((((((....)))))))	P
121	NM_003927:1600-1648	Homo sapiens methyl-CpG binding domain protein 2 (MBD2), transcript variant 1, mRNA	AGATGTATTTTTGATGTATATATCT<br>((((((((.........))))))))	P
200	NM_022763:6852-6894	Homo sapiens FAD104 (FAD104), mRNA	ATATTTATGCCCAATAAATGT<br>((((((((.....))))))))	P
150	NM_015578:2037-2116	Homo sapiens chromosome 19 open reading frame 13 (C19orf13), mRNA	TTTTATATAGTTGTAAAA<br>(((((((....)))))))	P, W
190	NM_145175:2274-2301	Homo sapiens NSE1 (NSE1), mRNA	AAAATTTCAAATTGAAATTTT<br>((((((((.....))))))))	P, W
227	NM_017893:4247-4275	Homo sapiens sema domain, immunoglobulin domain (Ig), transmembrane domain (TM) and short cytoplasmic domain, (semaphorin) 4G (SEMA4G), mRNA	ACAATGAATGTATTTATTGT<br>((((((((....))))))))	P, W

210	NM_005487:3043-3125	Homo sapiens high-mobility group protein 2-like 1 (HMG2L1), mRNA	AAATCTCTTAGATTT<br>(((((.....)))))	P, W
211	NM_014901:3848-3886	Homo sapiens ring finger protein 44 (RNF44), mRNA	ATGTATGTATTTGAGAAAATGCTAATATAT<br>((((((((((((....))))))..))))))	P
224	NM_020177:2578-2628	Homo sapiens fem-1 homolog c (C.elegans) (FEM1C), mRNA	AATATACCATATAATATATT<br>((((((........))))))	P
203	NM_002657:5587-5626	Homo sapiens pleiomorphic adenoma gene-like 2 (PLAGL2), mRNA	AATGAAGTTGTTTATT<br>((((((....))))))	P
35	NM_173822:2158-2178	Homo sapiens hypothetical protein MGC39518 (MGC39518), mRNA	TTTTGTTTAAAAACAAAA<br>(((((((....)))))))	P
197	NM_004703:3243-3262	Homo sapiens rabaptin, RAB GTPase binding effector protein 1 (RABEP1), mRNA	TTTATATTAAAATATGAA<br>(((((((....)))))))	P
27	NM_152267:3108-3127	Homo sapiens hypothetical protein FLJ38628 (FLJ38628), mRNA	ATTTTCACTGTTGTGAAAGT<br>((((((((....))))))))	P
239	NM_030797:1250-1337	Homo sapiens hypothetical protein DKFZp566A1524 (DKFZP566A1524), mRNA	ATGTTAATACTTGTGTATTTACAT<br>((((.(((((....))))).))))	P
262	NM_002819:1859-1920	Homo sapiens polypyrimidine tract binding protein 1 (PTBP1), transcript variant 1, mRNA	AAAGAGAAATCAGTTTACCTGTTTTT<br>(((((.....(((.....))))))))	P
256	NM_170677:977-1037	Homo sapiens Meis1, myeloid ecotropic viral integration site 1 homolog 2 (mouse) (MEIS2), transcript variant a, mRNA	TATCCGGACTGGGATA<br>(((((......)))))	P
200	NM_001677:1898-1994	Homo sapiens ATPase, Na+/K+ transporting, beta 1 polypeptide (ATP1B1), mRNA	TTTTTTCTGCAAGAAAAAG<br>(((((((.....)))))))	P
224	NM_014153:3602-3631	Homo sapiens zinc-finger protein AY163807 (HSPC055), mRNA	TTTAACACTAGTATTTGTTAAA<br>(((((((........)))))))	P
166	NM_005595:2467-2565	Homo sapiens nuclear factor I/A (NFIA), mRNA	TATCTTTGTAAGATA<br>((((((...))))))	P
246	NM_139135:7428-7485	Homo sapiens AT rich interactive domain 1A (SWI- like) (ARID1A), transcript variant 2,	GCAGCGGCTACGCTGC<br>((((((....))))))	P

		mRNA		
232	NM_015952:914-976	Homo sapiens RWD domain containing 1 (RWDD1), mRNA	TCAGGAGAATATTCTTCTGA<br>((((((((....))))))))	P
3	NM_005321:721-785	Homo sapiens histone 1, H1e (HIST1H1E), mRNA	AACCCAAAGGCTCTTTTCAGAGCCACCCA<br>........((((((....)))))).....	P
61	NM_020993:3639-3660	Homo sapiens B-cell CLL/lymphoma 7A (BCL7A), mRNA	AGATGAATTTGGATATTTATTT<br>((((((((......))))))))	W
178	NM_006480:2193-2215	Homo sapiens regulator of G-protein signalling 14 (RGS14), mRNA	GAGGAGGGGCCGGCCCCTCCTC<br>(((((((((....)))))))))	W
278	NM_007040:2857-2881	Homo sapiens E1B-55kDa-associated protein 5 (E1B-AP5), transcript variant 1, mRNA	GGGCTGCCTCCCTCCAGCCC<br>((((((........))))))	W
17	NM_004443:3616-3640	Homo sapiens EphB3 (EPHB3), mRNA	TCTTCATATTGAAGA<br>(((((.....)))))	W
221	NM_007373:2827-2873	Homo sapiens soc-2 suppressor of clear homolog (C. elegans) (SHOC2), mRNA	T-ATATATGTATATACAATGCTATATA<br>(.(((((.((((.....)))))))))))	W
214	NM_021190:2873-2928	Homo sapiens polypyrimidine tract binding protein 2 (PTBP2), mRNA	TTTTGAAATTGATGTACTTAGTTTCAAGATT<br>((((((((((((.....))))))))))))..	W
193	NM_016353:2379-2408	Homo sapiens zinc finger, DHHC domain containing 2 (ZDHHC2), mRNA	TTTAGTTTGAGATAAACTAAA<br>(((((((((...)))))))))	W
136	NM_005610:2031-2071	Homo sapiens retinoblastoma binding protein 4 (RBBP4), mRNA	GTAAAGATGTATGTTTTTAC<br>((((((((....))))))))	W
160	NM_001219:3240-3267	Homo sapiens calumenin (CALU), mRNA	TAGAGTGTAAACCAAGTTTTATATTCTG<br>(((((((((((......)))))))))))	W

167	NM_021038:4075-4159	Homo sapiens muscleblind-like (Drosophila) (MBNL1), mRNA	CATTAAAAGAACAAAGATCTTTTATATG<br>((((((((((.......))))))).)))	W
161	NM_001769:1038-1078	Homo sapiens CD9 antigen (p24) (CD9), mRNA	TTTTGCTTGTTATATTAAGCAGAA<br>(((((((((......)))))))))	W
246	NM_013365:2289-2308	Homo sapiens golgi associated, gamma adaptin ear containing, ARF binding protein 1 (GGA1), mRNA	GGGTGGGGTCTTCCCCACCT<br>((((((((....))))))))	W
71	NM_002078:7553-7578	Homo sapiens golgi autoantigen, golgin subfamily a, 4 (GOLGA4), mRNA	TTTTTCAGAGGAAAAA<br>((((((....))))))	W
21	NM_015355:3606-3643	Homo sapiens joined to JAZF1 (JJAZ1), mRNA	ATTCTTTATTTATAAAGGAT<br>((((((((....))))))))	W
251	NM_003244:1280-1308	Homo sapiens TGFB-induced factor (TALE family homeobox) (TGIF), transcript variant 4, mRNA	TGGAATACAGTCATTCCA<br>(((((......))))))	W
61	NM_006558:1749-1799	Homo sapiens KH domain containing, RNA binding, signal transduction associated 3 (KHDRBS3), mRNA	ATAGAATTTAGTTATTTTAT<br>(((((((......)))))))	W
186	NM_014583:1428-1451	Homo sapiens LIM and cysteine-rich domains 1 (LMCD1), mRNA	TTCTAAGAAGTCTTAGGA<br>(((((((....)))))))	W
	NM_002569:3320-3346	Homo sapiens furin (paired basic amino acid cleaving enzyme) (FURIN), mRNA	AGCCCGGGCTGCCTGGGCT<br>((((((((...))))))))	W
180	NM_005249:2317-2397	Homo sapiens forkhead box G1B (FOXG1B), mRNA	TGTATATTTTGATGTATG<br>(((((((....)))))))	W
11	NM_001546:1319-1388	Homo sapiens inhibitor of DNA binding 4, dominant negative helix-loop-helix protein (ID4), mRNA	CATCTATTGTTTAAAATAGATG<br>((((((((......))))))))	W
240	NM_005479:2438-2521	Homo sapiens frequently rearranged in advanced T-cell lymphomas (FRAT1), transcript variant 1, mRNA	ACACTTCGCACCGGAGTGT<br>((((((((...))))))))	W

232	NM_002265:3275-3300	Homo sapiens karyopherin (importin) beta 1 (KPNB1), mRNA	AGGCTAGAAGTAGCTT<br>((((((....))))))	W
65	NM_004429:2279-2308	Homo sapiens ephrin-B1 (EFNB1), mRNA	GTCGCGCCTCGTGCGGCA<br>(((((((...))))))).	W
182	NM_004343:1845-1899	Homo sapiens calreticulin (CALR), mRNA	CAAAATTTCTATTAAATTAAATTTTG<br>((((((((..........))))))))	W
214	NM_021190:2247-2290	Homo sapiens polypyrimidine tract binding protein 2 (PTBP2), mRNA	TTTTGAAATTGATGTACTTAGTTTCAAGATT<br>((((((((((((.....))))))))))))..	W
245	NM_130470:249-271	Homo sapiens MAP-kinase activating death domain (MADD), transcript variant 1, mRNA	CAGAATTCCTCCTGGGAATGCTG<br>(((.(((((.....))))).)))	W
225	NM_013381:3780-3801	Homo sapiens thyrotropin-releasing hormone degrading ectoenzyme (TRHDE), mRNA	AACTCATTTTCTTTGAGTT<br>((((((.......))))))	W
89	NM_006599:7764-7837	Homo sapiens nuclear factor of activated T-cells 5, tonicity-responsive (NFAT5), transcript variant 3, mRNA	GGAAATGGTATACTATTTT<br>.(((((((....)))))))	W
97	NM_032208:4055-4083	Homo sapiens anthrax toxin receptor 1 (ANTXR1), transcript variant 1, mRNA	TTGACTGCTGGCAGTCTAA<br>(((((((....))))).))	W

[1] Group ID is a serial number, which can be used to query the GLEAN-UTR database.

[2] “W” refers to the study by Washietl et al. and “P” to the one by Pedersen et al.
