# Supplementary material for: Mining small RNA structure elements in untranslated regions of human and mouse mRNAs using structure-based alignment
Source: BMC Genomics. 2008 Apr 25;9:189. doi: 10.1186/1471-2164-9-189 (PMC2413145; doi:10.1186/1471-2164-9-189)
Supplement: Additional file 5 — Extending RNA structure groups by PatSearch. The 90 structure groups were used to search human UTRs to obtain additional group members using PatSearch. GO analysis refers to filtering out hits without the same GO term annotation as the original group. The structure groups are ordered according to the difference between the original group size and the group size after PatSearch. [file 1471-2164-9-189-S5.pdf]

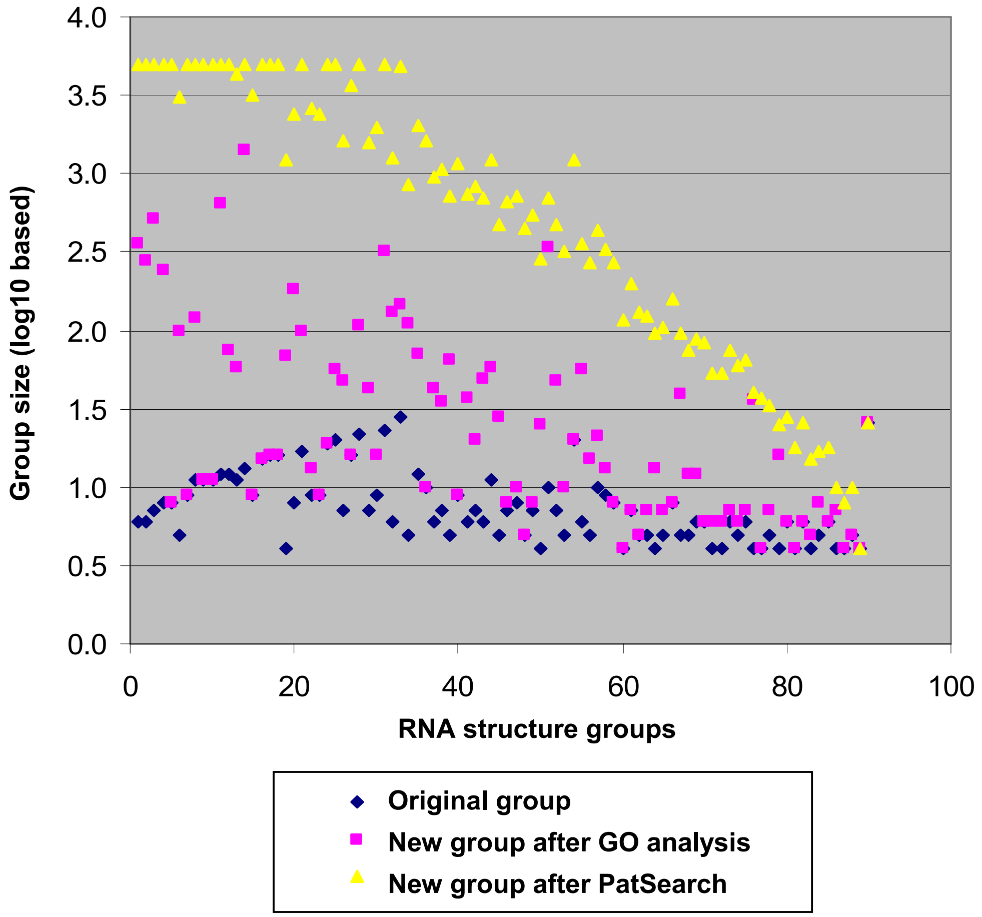

Additional file 5. Extending RNA structure groups by PatSearch. The 90 structure groups were used to search human UTRs to obtain additional group members using PatSearch. GO analysis refers to filtering out hits without the same GO term annotation as the original group. The structure groups are ordered according to the difference between the original group size and the group size after PatSearch.
